# Force Generation by Membrane-Associated Myosin-I

**DOI:** 10.1038/srep25524

**Published:** 2016-05-09

**Authors:** Serapion Pyrpassopoulos, Göker Arpağ, Elizabeth A. Feeser, Henry Shuman, Erkan Tüzel, E. Michael Ostap

**Affiliations:** 1 The Pennsylvania Muscle Institute and Department of Physiology, Perelman School of Medicine at the University of Pennsylvania, Philadelphia PA 19104-6085, USA; 2 Department of Physics, Worcester Polytechnic Institute, Worcester MA 01609, USA.

## Abstract

Vertebrate myosin-IC (Myo1c) is a type-1 myosin that links cell membranes to the cytoskeleton via its actin-binding motor domain and its phosphatidylinositol 4,5-bisphosphate (PtdIns(4,5)P_2_)-binding tail domain. While it is known that Myo1c bound to PtdIns(4,5)P_2_ in fluid-lipid bilayers can propel actin filaments in an unloaded motility assay, its ability to develop forces against external load on actin while bound to fluid bilayers has not been explored. Using optical tweezers, we measured the diffusion coefficient of single membrane-bound Myo1c molecules by force-relaxation experiments, and the ability of ensembles of membrane-bound Myo1c molecules to develop and sustain forces. To interpret our results, we developed a computational model that recapitulates the basic features of our experimental ensemble data and suggests that Myo1c ensembles can generate forces parallel to lipid bilayers, with larger forces achieved when the myosin works away from the plane of the membrane or when anchored to slowly diffusing regions.

Actin filaments are mechanically linked to lipid membranes by a variety of cytoskeletal, scaffolding, adaptor, signaling, and adhesion proteins^1^,^2^. Among these molecules are class-I myosins that bind directly to phosphoinositides and other anionic phospholipids through regions within their tail domains^3^,^4^,^5^,^6^,^7^,^8^. This important class of molecular motors participate in a number of dynamic membrane events, including endocytosis, exocytosis, membrane trafficking, ruffling, sensory transduction, cell shape control, and cell adhesion^8^. The role of the motor activity for all of these processes is not yet clear, but has been proposed to include powering vesicle motility, pulling and stabilizing of membrane tubules, movement or clustering of lipids or membrane-bound proteins relative to actin, or retraction of actin relative to membranes^8^,^9^.

To facilitate these roles, myosins may need to generate force on actin filaments oriented parallel to the plane of the membrane, pull on membranes to change their shape or form tubules along actin filaments, or transport lipids and associated proteins along actin filaments^8^,^9^. Myosin-I isoforms bind to lipid bilayers via the interaction with anionic phospholipids, with some isoforms having a preference for phosphoinositides^3^,^4^,^10^,^11^. The widely expressed isoform, Myo1c, binds phosphatidylinositol-4,5-bisphosphate (PtdIns(4,5)P_2_) with high affinity via a pleckstrin homology (PH) domain in its tail region, and membrane association via this domain has been shown to be necessary for the proper cellular localization of this isoform^3^,^10^.

Myo1c can power actin gliding in an *in vitro* motility assay when it is anchored via the PH-PtdIns(4,5)P_2_ linkage^12^. Actin-filaments can move over the fluid lipid bilayer at nearly the same rate as when the Myo1c is immobilized on a non-diffusive surface. Fluid bilayers are >100 times more viscous than water, so it is not surprising that membrane-bound myosin-I can drive the motility of unloaded actin filaments 12. However, the ability of membrane-bound myosin-I to generate forces against external resisting loads on actin filaments is still not known. Whether or not myosin can generate force parallel to the plane of the membrane depends on lipid membrane fluidity and on the effective diffusion constant of membrane-bound myosin. Lipids rapidly diffuse within the plane of the membrane, so individual lipids are not likely to be appropriate anchors for myosin-I to generate and sustain high values (>1 pN) of force, since any force would be rapidly dissipated into the fluid membrane during the lifetime of the actomyosin interaction. However, the collective behavior of multiple myosins bound to multiple lipids may lead to production of higher forces, or changes in membrane shape may facilitate tension maintenance and not dissipation, by allowing myosin to act away from the plane of the membrane.

To explore how fast a force applied to membrane-bound Myo1c dissipates in the fluid membrane, we used an *in vitro* single molecule level approach. We measured the time it takes for a force applied to a membrane-bound myosin to relax to zero due to diffusion within the plane of the membrane, and from that the effective diffusion coefficient (D) using Einstein’s relationship. Using an ensemble approach we also measured the ability of phosphoinositide-bound Myo1c to generate force relative to the membrane and displace actin filaments against external resisting load. To better understand our experimental system, and obtain additional insight into *in vivo* complexity not represented in our *in vitro* experiments, we also used computational approaches to explore the roles of diffusion and motor densities in modulating the ability of Myo1c to generate force relative to membranes, and to resist forces placed on the actin.

## Results

### Diffusion and drag coefficients of single membrane-bound Myo1c molecules

**Figure 1:**
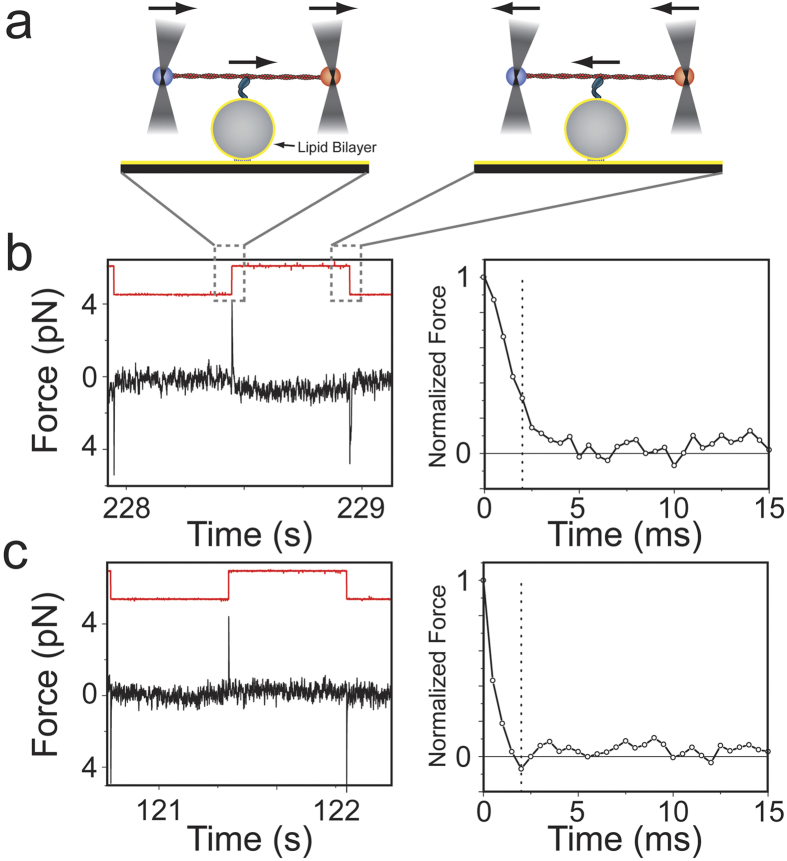
Relaxation of the force on an actin dumbbell held by optical traps undergoing a square wave oscillation. (**a**) Cartoon representation of the three-bead geometry. An actin filament held by two laser-trapped beads is oscillated over a spherical lipid coated pedestal while attached to Myo1c. (**b**), Left): Sample of the force trace for one of the two laser-trapped beads (black) and the square wave pulse (red; arbitrary units) which is applied to the laser traps, in the presence of 0.6 pM Myo1c and 1 μM ATP. The force offset due to pretention of the actin dumbbell (Methods) has been removed for the sake of clarity. (**b**), Right): Expanded view of the relaxation phase for a force spike from the left panel after normalization. The dashed vertical line indicates the 5^th^ point (2 ms) up to which the S_5_ value is calculated and is used as a first approximation of the rate of force relaxation (see text for details). (**c**) The same as in (**b**) but in the absence of Myo1c. (When the actin dumbbell is away from a lipid coated spherical pedestal data traces are similar to (**c**) and are therefore not presented here for this reason).

Using an optical trap, we measured the drag coefficient of PtdIns(4,5)P_2_-bound Myo1c in force-relaxation experiments. We applied abrupt changes in force to actin filaments to which membrane-bound Myo1c was attached, and then measured the time it takes the force to relax back to zero due to myosin’s diffusion on the membrane. Experiments were performed using single actin filaments suspended between two beads (actin dumbbell) held by separate optical traps that were brought close to the surface of a membrane-coated silica pedestal bead (Fig. 1). Pedestal beads (5 μm diameter) were coated with lipid membrane consisting of 2% PtdIns(4,5)P_2_, 2% BiotinylPE, and 96% DOPC and attached via streptavidin to a supported lipid bilayer on a coverslip of the same lipid composition (Methods). Efficient coating of pedestals with a fluid membrane was confirmed by confocal microscopy (Supplementary Movie 1) and fluorescence-recovery-after-photobleaching (Supplementary Movie 2). The positional stability of the lipid-coated pedestals was similar to nitrocellulose-coated pedestals immobilized using conventional attachment strategies (Supplementary Information & Supplementary Fig. 1).

To abruptly increase the force on the actin filament, a 110 ± 26 nm amplitude square-wave oscillation of 1 Hz was applied to the position of the optical traps holding the dumbbell, and the relaxation time of the actin dumbbell back to its equilibrium state (i.e., zero force) was measured for every oscillation cycle. The relaxation time is determined by the drag coefficient of the dumbbell. When the actin dumbbell is not bound to the lipid membrane, the drag coefficient is largely defined by the aqueous environment (i.e., hydrodynamic forces on the beads and the actin filament). However, when actin is bound to membrane via Myo1c, the drag coefficient increases due to viscous forces that Myo1c experiences due to diffusion in the membrane. Measurements were made with the actin filament adjacent to lipid-coated pedestals in the presence of Myo1c in the final assay buffer or experimental chamber (Fig. 1a,b), away from lipid coated spherical pedestals, and in contact with a lipid coated spherical pedestal in the absence of Myo1c (Fig. 1c). To ensure that myosin remains attached to actin during the time period of an oscillation, (~1s = T_oscillation_), a low ATP concentration (1 μM) was used to increase the average actomyosin attachment duration (3.8 s in the absence of force)^13^.

**Figure 2:**
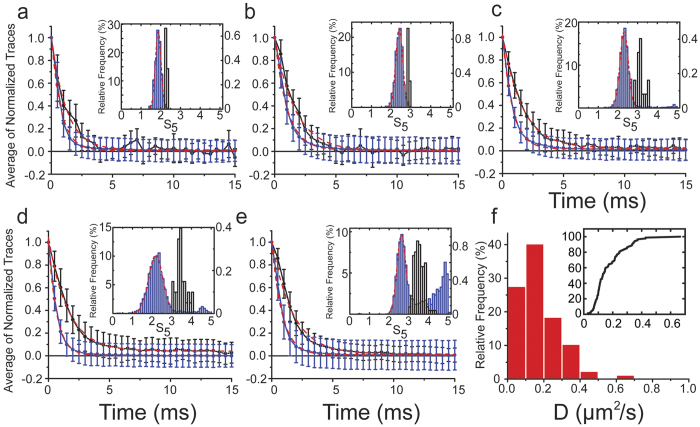
Measurement of viscous drag of membrane-bound Myo1c. Ensemble averages of normalized force relaxation traces obtained from oscillating actin filaments (**a**) away from the pedestal surface and (**b**) touching the top of the pedestal in the absence of Myo1c, and adjacent to the pedestal in the presence of (**c**) 0.2 pM, (**d**) 0.6 pM, and (**e**) 1.9 pM of Myo1c. The black and blue traces are averages of normalized force relaxation traces in the presence and the absence of actoMyo1c-membrane attachment respectively according to the parsing criterion (see text). Dashed red lines are error-weighted fits of the corresponding average traces to a single exponential decay function. Error bars are standard deviations of the mean. (Insets, blue bars, left axis) Frequency distribution of S_5_ values for all relaxation traces from a single experiment over multiple oscillation cycles (for the total percentage of the different types of relaxation over the whole datasets under each condition see Table 1). (Black bars, right axis) Distribution of S_5_ values identified as exponentially decaying force relaxation events during an actoMyo1c-membrane attachment. Blue bars to the left of black bars correspond to relaxation events in the absence of actoMyo1c-membrane attachment. Dashed red lines are fits of the predominant peaks (blue bars to the left of the black bars) to a Gaussian function. (**f**) Distribution of the diffusion coefficients (*D*_*Myo1c*_) obtained for membrane-bound Myo1c from fitting the individual force relaxation traces for actoMyo1c-membrane attachments identified in the presence of 0.2 pM and 0.6 pM Myo1c (see text for details). (Inset) The same data plotted as a cumulative distribution.

Examples of raw data and normalized traces of force relaxation events for different experimental conditions are shown in Fig. 1. To identify relaxation events that occurred during actoMyo1c-membrane attachment, we calculated for each normalized relaxation event the sum of the five points (S_5_) acquired during the first 2 ms after the force change imposed by the square-wave oscillation (Fig. 1b,c), which approximates the area under the force trace. When Myo1c is not included in the final assay buffer, the distributions of S_5_ values over multiple oscillation cycles acquired with the actin away from (Fig. 2a) or touching the pedestal (Fig. 2b) are predominantly unimodal and symmetric and can be described by a Gaussian distribution (red dashed lines in Fig. 2 inserts). In the presence of Myo1c, most relaxation events had S_5_ values within the control distribution, but the values beyond the tail of the control distribution increased with Myo1c concentration (Fig. 2). Traces that had S_5_ values >2.5 standard deviations over the mean of the predominant population in each experiment were defined as relaxation events occurring during actoMyo1c-membrane attachment. In the absence of a pedestal, or when the dumbbell is in contact with the lipid membrane in the absence of Myo1c, fewer than 0.5% of the events met this criterion (Fig. 2, Table 1).

Averages of the normalized relaxation force traces in the absence of identifiable actoMyo1c-membrane attachment (S_5_ ≤ μ ± 2.5·σ) from the five different conditions were well fitted by a single-exponential decay function (*τ*_*dbbl*_ = 0.62 − 0.82 ms; gray traces in Fig. 2a–e). Lifetimes obtained from the fits are close to the theoretically estimated lifetime of relaxation (*τ*_*dbbl*_ = 0.91 − 1.4 ms) assuming a simple viscoelastic model for the relaxation of the actin dumbbell under the laser-trap restoring force (Supplementary Information):


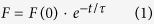


where *τ* = γ/*k* is the relaxation lifetime, *k* is the elasticity of the trap (assuming that it is much more compliant than any other element of the dumbbell), and *γ* is the drag coefficient of the actin dumbbell in aqueous environment. The fitting parameters as well as the number of events for each average trace are summarized in Table 1.

**Figure 3:**
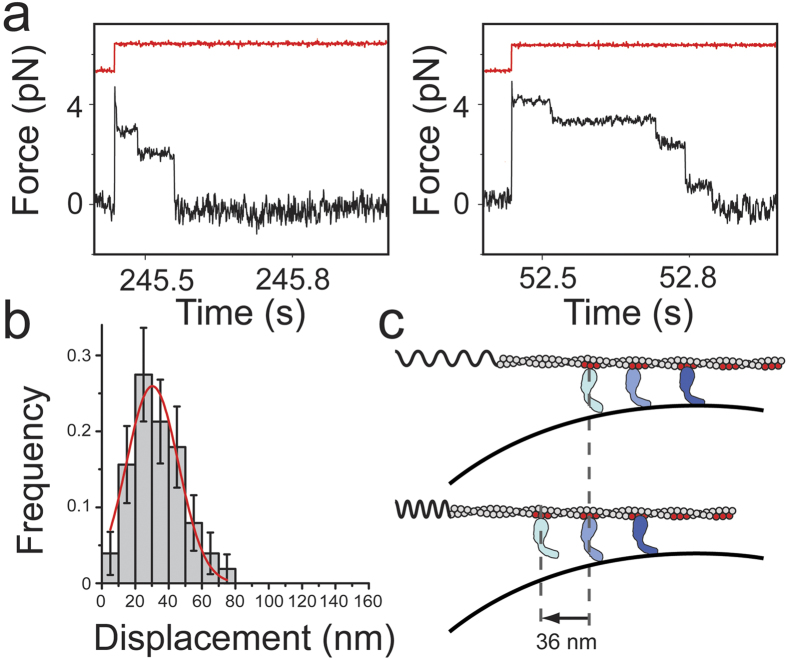
Stepwise relaxation of the force on an actin dumbbell oscillating under a square wave pulse (1 μM ATP). (**a**) Two examples of stepwise relaxation events (black trace) that occurred in the presence of 0.6 pM Myo1c. Square wave pulses are shown with arbitrary units (red traces). (**b**) Frequency distribution of the displacements of the stepwise relaxations acquired in the presence of 0.2 pM Myo1c. Changes in force were transformed to displacement traces (Methods). Error bars were estimated from 1000 bootstrap cycles and the red trace is an error-weighted fit of the distribution to a Gaussian function. (**c**) Cartoon representation of the proposed origin of the stepwise relaxation events. The motion of an actin filament to its equilibrium position under the restoring force of the laser-trap (stretched spring in upper subpanel) is hindered by a Myo1c molecule (light blue color) which cannot be dragged further to the left due to the curvature of the underlying pedestal. When the Myo1c molecule detaches from the pedestal (lower subpanel) the actin filament moves towards the left until another Myo1c molecule (pale blue) reaches the same geometrical spot. The size of the sequential actin displacements is determined by the spacing between Myo1c molecules (red colored spots on actin filament) which can interact simultaneously with the actin filament and the lipid coated pedestal. This spacing according to structural studies comes in integer multiples of ~36 nm.

Force relaxation events defined as occurring during actoMyo1c-membrane attachment (S_5_ > μ ± 2.5·σ) decreased either exponentially (Fig. 2) or in a stepwise manner (Fig. 3, Supplementary Fig. 2). The percentage of both classes increased with increasing Myo1c concentration (Table 1). The total number of relaxation events that were defined as actoMyo1c-membrane attachments (S_5_ > μ ± 2.5·σ) in the presence of 0.2 or 0.6 pM Myo1c were less than 10% of the total cycles (Table 1) and were in the realm of single-molecule events (Supplementary Fig. 3). Those that did not exhibit step-wise, but rather exponential behavior represented less than 2% of the oscillation cycles (Table 1) and the corresponding average traces were well fitted by single-exponential decay functions (Fig. 2c,d) with lifetimes (*τ*_*total*_ = 1.6 ms) greater than found in the absence of actoMyo1c-membrane attachments. The drag coefficient (*γ*) of the actin dumbbell is expected to increase due to the additional drag of the membrane-bound Myo1c along the surface of the fluid membrane as the dumbbell relaxes back to its equilibrium position. The spring constant *k* is assumed to remain unaffected since the trap stiffness (0.02–0.03 pN/nm) is at least 10-fold lower than the myosin stiffness^14^. Assuming the drag coefficients of the actin dumbbell (*γ*_*dbbl*_ = *τ*_*dbbl*_·*k*) and membrane-bound Myo1c (*γ*_*Myo1c*_ = *τ*_*Myo1c*_·*k*) are additive, one gets the drag coefficient as well as the relaxation lifetime of membrane-bound Myo1c (Table 1):


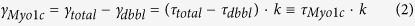


The diffusion coefficient *D*_*Myo1c*_ was calculated by the Einstein relationship


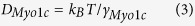


(*D*_*Myo1c*_ = 0.16 μm^2^/s; Table 1) using the lifetimes from the exponential fits of the averaged relaxation traces (Fig. 2, Table 1). A distribution of diffusion coefficients from the fits of individual relaxation traces was also obtained (Fig. 2f), and the average value was calculated (*D*_*Myo1c*_ = 0.17 ± 0.11 μm^2^/s). Using total-internal reflection fluorescence (TIRF) microscopy, we also measured the distribution of diffusion constants of Myo1c molecules bound to short fluorescent-actin filaments and bound to planar, fluid, lipid bilayers with similar composition (Supplementary Information). The distribution of the calculated diffusion constant values from the TIRF data is broad, and we are not able to rule out the possibility that the distribution contains events with more than one myosin bound to the actin filaments. Nevertheless, the values determined using the laser trap are within the distribution of diffusion constants measured by TIRF in the absence of force (Supplementary Fig. 4).

We next investigated the origin of the stepwise decreases in force observed in the force-relaxation experiments (Fig. 3) by determining the forces (Fig. 3, Supplementary Fig. 5) and dwell times of the constant force regions of the traces. Because we know the optical trap stiffness, we can determine the size distributions of the displacement steps (Methods). In the presence of 0.2 pM Myo1c, the distribution exhibits a predominant peak at ~30 nm, which is similar to the spacing between target zones along the long-helical axis of the actin filament for membrane-bound Myo1c (Fig. 3)^15^. In support of this interpretation, a second peak in the distribution of ~60 nm is observed at a higher Myo1c concentration (60 pM; Supplementary Fig. 5). We interpret this ~30 nm periodicity to reflect the forcible detachment of actin-bound Myo1c that has been dragged or diffused to a boundary of the spherical pedestal during the oscillation (Fig. 3c). The geometrical boundaries of the pedestal are functioning as diffusion barriers beyond which Myo1c molecules cannot be pulled any further along the direction of oscillation until they detach either from the membrane or the actin.

The average duration times of the steps during the stepwise relaxations for the three different Myo1c concentrations were 38 ± 69 ms (0.2 pM), 47 ± 81 ms (0.6 pM) and 73 ± 110 ms (1.9 pM) (errors are standard deviations). These durations are substantially shorter than the expected average actoMyo1c attachment duration in the presence of 1 μM MgATP, which is 3.8 s in the absence of force^13^ and likely represent the detachment of Myo1c from the lipid bilayer. The attachment duration of Myo1c to PtdIns(4,5)P_2_ is known to be force sensitive and is expected to be between 14–500 ms for the range of forces observed in the stepwise relaxation traces (≤5 pN)^16^. For the same type of experiment using a non-diffusive biotinylated construct of Myo1c (Myo1c^3IQ^) anchored via streptavidin on nitrocellulose-coated spherical pedestals single-molecule actomyosin attachments were in the order of seconds to hundreds of seconds. We therefore exclude the possibility that the stepwise interactions are due to Myo1c molecules bound to spots of defective lipid coating.

### Actin gliding powered by ensembles of membrane-bound Myo1c under resisting load

**Figure 4:**
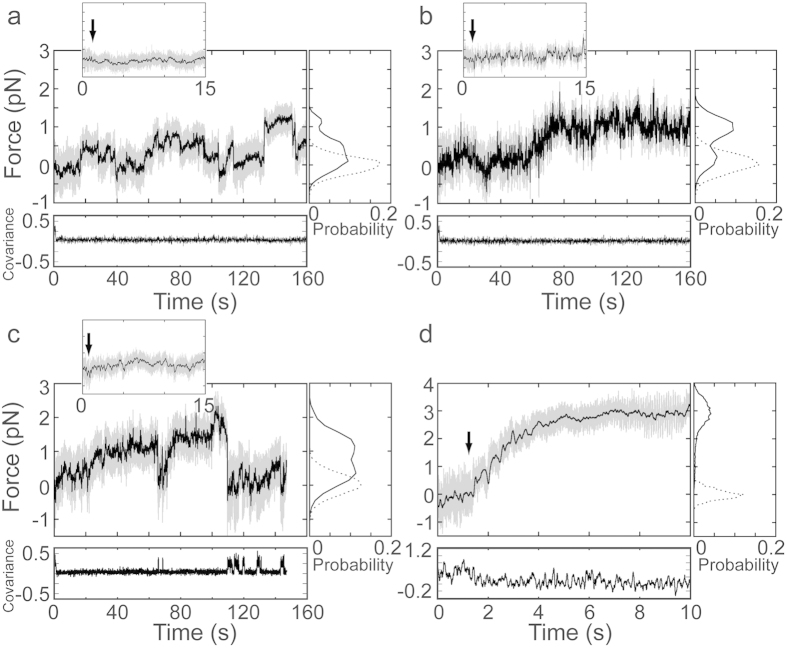
Force developed on actin filaments by ensembles of Myo1c molecules on lipid and solid substrates (1mM ATP). (Large panels) Force on actin dumbbells pulled by Myo1c molecules bound to lipid coated spherical pedestals in the presence of (**a**) 15 nM and (**b**) 150 nM Myo1c. Actin dumbbells pulled by Myo1c^3IQ^ molecules anchored via biotin-streptavidin linker on nitrocellulose-coated spherical pedestals in the presence of (**c**) 18 nM and (**d**) 360 nM Myo1c. Unfiltered (gray) and smoothed (black) force traces of the beads attached to the barbed-end of the actin filaments are shown. (Insets) Expanded views of the first 15 s of the force traces, with the black arrows indicating the instant of attachment of pedestal-bound Myo1c molecules to the actin filament. (Right subpanels) Probability histograms of the force before the initial attachment (dashed lines) and after the initial attachment (solid lines). (Lower subpanels) Covariance traces (×500) of the two laser-trapped beads. Low covariance values indicate attachment of the actin filament to pedestal-bound Myo1c molecules, while high covariance values indicate detachment.

We measured the ability of ensembles of membrane-bound Myo1c to generate force against external resisting load using the three-bead assay. The laser traps in this case were not oscillated, but were rather used to measure the force generated by pedestal-attached Myo1c molecules. Attachment of actin dumbbells to lipid-coated pedestals was measured in the presence of Myo1c and 1 mM MgATP. Myo1c was added directly to the final assay buffer, allowing dynamic attachment to PtdIns(4,5)P_2_-containing membranes^12^. Actin-pedestal attachments were identified by the drop in the force covariance values of the trapped beads (Fig. 4; Methods; ref. 17). We observed long interactions (>100 s) with forces that fluctuated between 0–1 pN in the presence of 15 nM Myo1c. The average value of force from 5 pedestals was 0.47 ± 0.22 pN. Although the force was variable, the covariance of the trapped beads remained low indicating that the actin dumbbell remained attached to the membrane throughout the event^17^,^18^. Therefore, at this concentration, membrane-bound Myo1c can develop forces as high as 1 pN but cannot sustain them for extended periods of time (>5 s). Average actin attachment durations of single Myo1c interactions in the presence of 1mM ATP are expected to be ~250 ms^13^, so the observed attachments were the result of many actomyosin interactions. Increasing the Myo1c concentration to 150 nM decreased the amplitude of the force fluctuations. The higher concentration did not increase the maximum force observed, but rather the length of time that the high forces can be sustained (~1 pN; Fig. 4b). The average force obtained from three different actin dumbbells in different chambers at 150 nM Myo1c is 0.86 ± 0.53 pN. Forces were not developed and pedestal-actin attachments were not observed (as determined by drops in covariance values) when dumbbells were brought into contact with pedestals in the absence of Myo1c or in the presence of membranes without PtdIns(4,5)P_2_.

Force development was also measured using a biotinylated Myo1c construct (Myo1c^3IQ^; Methods) that was firmly attached to streptavidin adsorbed to nitrocellulose-coated pedestals^13^. When the Myo1c^3IQ^ concentration was adjusted to attain interaction forces similar to those observed with membrane-bound Myo1c (~1 pN; Fig. 4c), the calculated force covariance of the beads was found to vary between low and high levels that correspond to bound and detached states. This behavior is unlike the continuous attachment observed on membrane-coated pedestals (Fig. 4a,b), and it may reflect the inability of the surface anchored motors to change their spatial position to accommodate shifting in the position of the binding target zones along the actin^15^. Notably, when the Myo1c^3IQ^ concentration was increased to obtain continuous attachment of the actin dumbbell to the pedestal, linear actin displacements were observed that persisted at forces greater than observed on membranes (>2.5 pN; Fig. 4d).

### Computational Modeling of Membrane-Bound Myo1c Motility

To gain insight into the mechanism of force generation on fluid bilayers, and to understand the motile behavior of ensembles of Myo1c motors, we developed a one-dimensional coarse-grained mesoscale model of actin motility with Myo1c either bound rigidly to a surface or attached to a fluid lipid-bilayer (Supplementary Figs 6 and 7). The motors and the actin dumbbell are subject to Brownian forces, where the latter also feels the force from the optical trap. Diffusion coefficients of the actin dumbbell are obtained from experiments (Methods). In the membrane-based model, Myo1c molecules bind and unbind to the actin based on experimentally determined rates (Table 2) and exert forces while diffusing on the substrate using diffusion coefficients pulled stochastically from the experimentally determined distribution (Fig. 2f).

**Figure 5:**
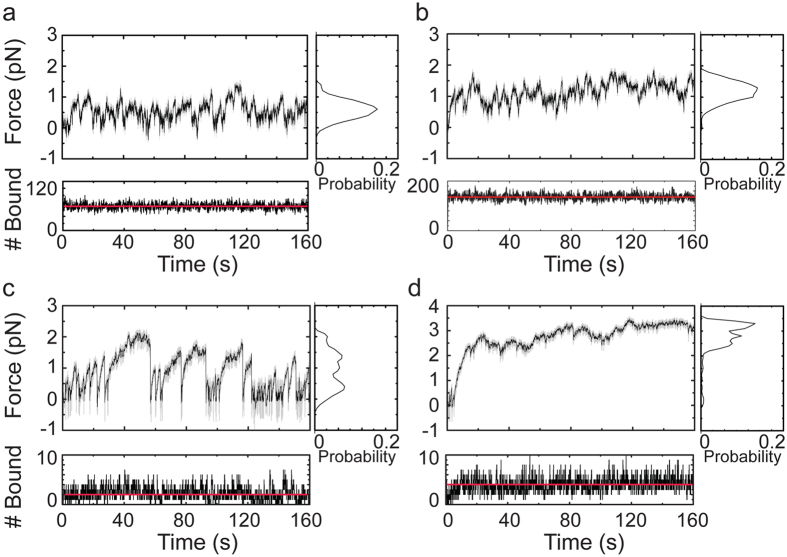
Simulated force developed on actin filaments by ensembles of Myo1c molecules on lipid and solid substrates. (Large panels) Force on actin dumbbells pulled by Myo1c molecules dynamically attached to a diffusive surface at densities corresponding to average number (over 50 realizations) of actin-bound motors (**a**) N = 69.04 and (**b**) N = 123.61. Actin dumbbells pulled by Myo1c^3IQ^ molecules rigidly anchored to a surface at densities corresponding to average number of actin-bound motors (**c**) N = 2.14 and (**d**) N = 3.56. Unfiltered (gray) and smoothed (black) force traces of the beads attached to the barbed-end of the actin filaments are shown. (Right subpanels, dashed lines) Probability histograms of the force are also shown. (Lower subpanels) Traces showing the number of Myo1c molecules bound to actin filament as a function of time.

**Figure 6:**
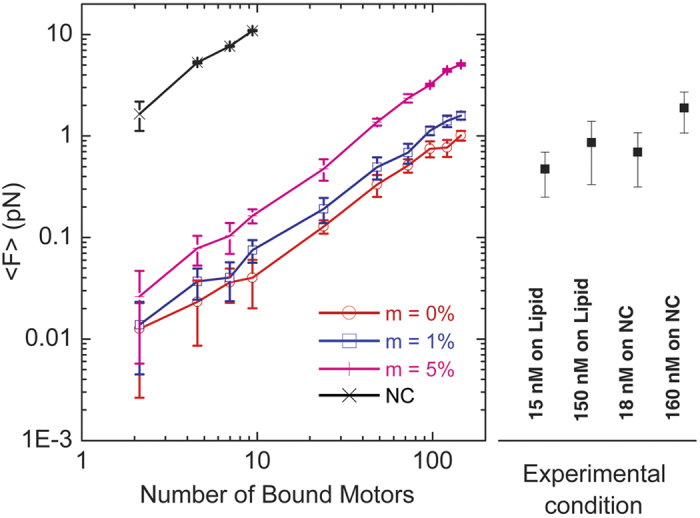
Average forces on actin filaments as a function of the number of motors bound to the filament. (Left) The average force <*F*> (over 10 realizations) of the traces of the beads attached to the barbed-end of the actin filaments as a function of the average number of actin-bound motors, N, on a diffusive surface obtained from the simulations. An increasing percentage of non-diffusing motors (m = 1%, 5%) that dynamically interact with the surface results in an increase in the average force. The dependence of the average force on the number of motors when they are rigidly attached to a non-diffusing nitrocellulose (NC) surface is also shown. Error bars show standard deviation. (Right) Average force values and standard deviations obtained from experimental data at the indicated protein concentrations are shown.

As an initial test of our model, we simulated unloaded motility in the absence of forces imposed by the optical trap. The actin gliding speed was found to be ~40 nm/s and 42 nm/s for nitrocellulose and lipid coated pedestals, respectively (Supplementary Fig. 8), which is within 2-fold agreement with experimental results^12^. We also measured the mean squared displacement (MSD) of the actin dumbbell in the absence of myosin motors and the trap, and the MSD of free myosin motors on the lipid coated surface (Supplementary Fig. 8), and obtained excellent agreement with experiments. We then performed simulations over a range of number of myosins bound to 705 nm long pedestals on which they were allowed to diffuse or were rigidly attached (Supplementary Fig. 6–7, Supplementary Movies 3, 4, 5, 6) in the presence of an actin dumbbell being held in an optical trap. We quantitatively compared the mean trap forces upon reaching steady-state (Fig. 5), and identified the average number of motors (average number of motors per pedestal or equivalently number of actin-bound motors) for which force values matched experimental observations (Fig. 6). The trajectories of the simulated force traces of the actin dumbbell interaction with lipid coated pedestals at these numbers of motors have similar characteristics as the experimental traces (Figs 4 and 5). To achieve forces and deviations similar to the 15 nM conditions (low density) an average of ~69 myosins bounds to actin were required. At the 150 nM conditions, ~124 myosins bounds to actin were required.

To achieve forces and deviations similar to the 18 nM conditions bound to nitrocellulose the required number of motors is substantially lower than on lipid. As found in the experiments, simulated force traces of Myo1c bound to nitrocellulose-coated pedestals vary in the 0–2 pN range, with periods of complete detachment (Fig. 5c), which is due to the very low number of attached motors (Table 3). At higher number of motors, the actin dumbbell remains bound to the pedestal while the force linearly increases to a stall force of ~3 pN (Fig. 5d), as seen in the experimental data (Fig. 4d).

**Figure 7:**
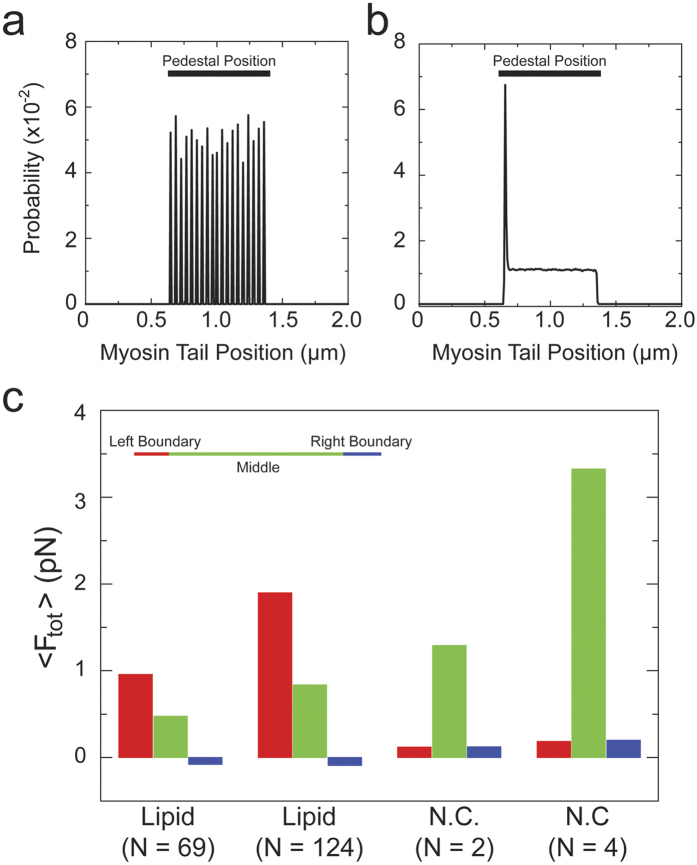
Maximum forces on surface-attached myosins as a function of position on pedestal. Position histograms of the maximum force on Myo1c motors as a function of their tail position on simulated (**a**) nitrocellulose- and (**b**) lipid-coated pedestals. The average number of actin-bound motors in (**a**) N = 3.56 and (**b**) N = 123.61. Maximum forces are recorded at every simulation time step, and the resulting histograms are averaged over 50 realizations. (**c**) The total average force, <*F_tot_*> generated in three different regions on the pedestal by myosin motors for different substrates and different average number of motors. The *left* and *right* boundary regions correspond to the first 8 nm from the outermost points of the pedestal, and the *middle* region in between the two boundaries (the inset is not to scale). The average force (<*F_tot_*> in Table 3) in a given region is calculated from the product of the average number of bound motors to actin and the mean average force of a single myosin during *τ*_on_ (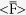 in Table 3, also see Supplementary Information).

Simulations revealed a relationship between the position of the myosin on the lipid substrate and the maximum force on the myosin during actin attachment (Fig. 7a,b). The maximum force exerted by a myosin bound to the non-diffusing substrate at a given time is evenly distributed across the surface (Fig. 7a). However, the probability of the maximum force for a lipid-bound myosin is substantially larger on the side of the substrate near the barbed-end of the actin dumbbell than the rest of the surface (Fig. 7b). Another useful force is the average force exerted by a myosin during one cycle. Quantification of this force, normalized for the number of myosins in an 8 nm (the size of the Myo1c working stroke^13^) boundary at the left-side (barbed end), indicates that ~71% of the force felt by the trap is generated by the ~5% of the myosins within this region (Fig. 7c, Table 3) when the average number of actin-bound motors is 69. This increased force per myosin is due to the geometric constraint that prevents the leftward diffusion of the myosin at the edge of the pedestal towards the barbed end. Interestingly, the simulation likely underestimates the force contribution compared to the experiment, since more motors will be expected to accumulate near the boundaries due to the curvature of the spherical pedestal.

We computationally examined the effect of including a small population of non-diffusing motors into the simulations on lipid surfaces (Fig. 6). As expected, inclusion of non-diffusing motors substantially increases the average force felt by the optical trap. For example, inclusion of 5% non-diffusing motors increases that average force >5-fold (Fig. 6).

## Discussion

Our goal was to measure directly the range of forces that myosin-I can generate force while bound to a fluid lipid bilayer. Vertebrate myosin-I isoforms bind to cellular compartments that have varying geometries, including tubulovesicular organelles, vesicular cargos, and the plasma membrane, which includes membrane protrusions and invaginations. Therefore, we evaluated force generation by Myo1c that was bound to a spherical-supported-bilayer tightly adhered to a coverslip, which provides a surface over which myosin can apply forces parallel to the plane of the membrane while interacting with actin, but will also apply forces away from the plane parallel to the membrane due to the pedestal curvature (Fig. 3c).

Direct measurements made with the optical trap show that forces on single Myo1c motors bound to PtdIns(4,5)P_2_ containing membranes relax to zero with a lifetime of 1 ms, which is 100-fold shorter than the lifetime of the force-bearing actoMyo1c state (~250 ms^13^) populated during steady-state ATPase cycling. This rapid relaxation time agrees well with the rate predicted by calculations using the diffusion constant obtained from TIRF tracking experiments in the absence of external force (Supplementary Information). The diffusion coefficient values for membrane-bound Myo1c in our model system are in the same range for diffusion constants of lipids in plasma membrane^19^ or fluorescent constructs of phosphoinositide binding proteins in living cells^20^,^21^. These values are approximately 10-fold lower than the reported diffusion coefficient of PtdIns(4,5)P_2_ within supported lipid bilayers^22^. However, Braunger *et al*.^22^ showed that different SUV spreading conditions used to form supported lipid bilayers can lead to formation of PtdIns(4,5)P_2_ clusters, which have lower diffusion coefficients, and Seu *et al*.^23^ demonstrated that different treatments of the same solid-substrate surface can lead to 3-fold differences in diffusion coefficients for supported bilayers of the same composition. Importantly, the inclusion of Mg^2+^ in assay buffers (which is crucial for the enzymatic activity of Myo1c) has been shown to induce PtdIns(4,5)P_2_ clusters that have a 4-fold reduction in the diffusion coefficient^24^. Finally, Myo1c interacts with more than one PtdIns(4,5)P_2_ molecules via a specific and non-specific binding sites^25^, which may result in slower diffusion. Any or all of the above contributions may be responsible for the low diffusion coefficient values of membrane bound Myo1c in our model system. Despite the range of diffusion constants, our results indicate that any forces exerted on actin by low concentrations of membrane-myosin will rapidly dissipate in the membrane by diffusion.

Increasing the Myo1c concentration to the point that the actin filament remains bound to the pedestal for long time periods resulted in fluctuating values of force that reach ~1 pN (Fig. 4b). The maximum force was not substantially increased with increasing Myo1c concentration, but the ability to sustain the maximum force for extended period of times was increased. Thus, the collective behavior of membrane-bound myosin ensembles, while not able to generate forces and displacements equivalent to motors rigidly bound to a pedestal (Fig. 4d), can exert forces and generate power relative to a fluid bilayer.

Our computational model provides possible mechanisms for force generation by membrane bound Myo1c and estimations of the Myo1c densities required to generate the experimentally observed forces (Figs 5 and 6). The average value of the number of actin-bound motors required in our simulations to develop and sustain forces of ~1 pN on lipid bilayers was ~124 at any given moment. Given the long-axis helical pitch of actin, myosins can bind simultaneously to actin and the membrane at approximately every 30–39 nm^15^. Notably, our oscillation experiments show clear evidence of this periodicity of target zones. If one assumes that 3–4 myosins can bind in every target zone^26^, one would expect about 54–94 myosins for 705 nm of actin. Given the fact that the simulations do not take into account any potential steric hindrances due to the high number of motors, and the reduced boundary effects due to a simplified one-dimensional pedestal, the agreement with the simulations is reasonable. It is also important to note that according to our computational model, any reduction in the pool of motile motors (either due to crowding or even lower diffusion coefficient motors) will considerably reduce the number of actin bound motors required to match any given value of force. For instance, if in simulations we assume 1% non-diffusive motors, then the maximum number of motors required to develop and sustain forces of ~1 pN for extended time periods on lipid bilayers is 40 instead of the 124 motors on actin at any given moment.

Our computational model predicts that approximately 35% of the force on the actin filament originates via the collective action of 92% of the attached myosins which generate ~0.007 pN per molecule. The remaining force comes from a small percentage of the myosins at the geometric boundary of the pedestal (Table 3). These myosins generate force against the diffusive boundary, such that forces would pull away from the plane of the membrane. Experimental evidence for these adhesive forces is clearly observed in the stepwise detachment events recorded in the oscillation experiments at increasing myosin concentration (Fig. 3), which are similar in magnitude and lifetime as we measured previously^16^.

### Force Generation by Cellular Myosin-I

Our experiments suggest that high densities of myosin-I isoforms can generate and sustain sub-pN forces parallel to the plane of a fluid lipid bilayer. These forces may be important for putting tension on polymerizing actin filaments that are driving membrane protrusion or drive the formation and secretion of vesicles from the tip of the microvilli^8^,^27^,^28^,^29^,^30^. It is also clear that myosin can generate higher forces in a direction away from the plane of the membrane, which is due to the adhesive interaction between the positively charged tail domain and anionic head groups of PtdIns(4,5)P_2_^16^. These forces likely play a role in the tubulation of lipid membranes and the transport and anchoring of vesicular cargos^31^,^32^,^33^,^34^,^35^,^36^,^37^.

The experimental assay used in these studies does not take into account the added complexity of myosin-I binding proteins (e.g.^38^,^39^,^40^,^41^,^42^,^43^,), lipid domains, or other constraints found in the cell that can lower the effective diffusion constant of membrane-bound myosin. Our simulations suggest these factors can have substantial effects on force generation. Indeed, introduction of a small fraction of non-diffusing motors results in substantial increase of the average force in the simulated traces (Fig. 6). These results strongly suggest that molecular crowding and entanglements play a role in the value of forces generated, particularly at high densities of motors, and that binding to less diffusive receptors or adhesion proteins can help in generating and sustaining higher forces by providing anchor points. Future experiments will be required to evaluate the role of myosin-I-binding proteins in altering membrane diffusion and adhesion lifetime.

## Methods

### Reagents

*In vitro* lipid cargo motility assays and laser trap experiments were performed in KMg25 (60 mM MOPS, pH 7.0, 25 mM KCl, 1 mM DTT, 1 mM EGTA, 1 mM MgCl_2_). Actin was purified^44^ and polymerized in KMg25 buffer and stabilized with Alexa Fluor 488 phalloidin (Invitrogen). 1,2-dioleoyl-*sn*-glycero-3-phosphocholine (DOPC), L-α-phosphatidylinositol-4,5,bisphosphate (PtdIns(4,5)P_2_), 1,2-dioleoyl-*sn*-glycero-3-phosphoethanolamine-N-(lissamine rhodamine B sulfonyl) (Liss-Rhod PE) and 1,2-dioleoyl-*sn*-glycero-3-phosphoethanolamine-N-(biotinyl) (Biotin-PE) were purchased from Avanti Polar Lipids. Silica beads (5 μm dia.) were purchased from Polysciences and 0.54 μm dia. beads were purchased from Bangs Laboratories. Streptavidin was purchased from Life Sciences.

### Protein Expression and Purification

Full length Myo1c (Myo1c) with a C-terminal Flag tag for affinity purification was coexpressed with calmodulin in Sf9 cells and purified as described^12^. Biotinylated mouse myo1c construct consisted of the motor domain, regulatory domain with three IQ motifs, a C-terminal FLAG tag and a C-terminal AviTag for site-specific biotinylation (Myo1c^3IQ^) was expressed and purified^45^. Calmodulin was prepared as described^46^. The actin-binding domain of human α-actinin-1 was expressed and purified as described previously^13^. A gelsolin construct truncated after the first domain that caps F-actin without severing activity^47^ was a gift from Dr. Daniel Safer. Purified proteins were flash-frozen in liq. N_2_, and stored at −80 °C in KMg25.

### Preparation of spherical and planar supported lipid bilayers (SLBs)

Small unilamellar vesicles (SUVs) for formation of supported lipid bilayers were prepared as described previously^16^,^48^ with modifications. In brief, lipids were mixed in chloroform at the appropriate molar ratios, dried under vacuum, and dissolved to 2 mg/mL in HNa100 (10 mM HEPES pH 7.0, 100 mM NaCl, 1mM DTT, 1mM EGTA) with vortexing at maximum speed for 2 min. Lipid solutions were subjected to four freeze-thaw cycles followed by extrusion through 30 nm pore membranes 11 times. SLBs were generated by washing 40 μl of silica beads (9.92% solid) as follows: 1 × Methanol, 1 × 1mL 1N KOH, bath sonicated for 15 min (in KOH), and 7 × 1mL water. Each wash was followed by spinning at (800 × g) for 2 min at room temperature. After the final washing, pelleted beads were mixed with 600 μl freshly made SUVs. The mixture was vortexed briefly and incubated overnight at room temperature. The following day, the beads were washed 3-times in 1 mL HNa100. Beads were kept in 0.5 mL HNa100 at room temperature and used for no more than 2 days. Planar supported lipid bilayers (SLBs) were made as described previously^12^.

### Confocal Imaging of Spherical Supported Bilayers and Fluorescence Recovery after Photobleaching

Imaging of fluorescent spherical supported bilayers containing 0.1% LRPE – 2% PtdIns(4,5)P_2_ – 97.9% DOPC was performed at room temperature using a 561-nm laser on a spinning-disk confocal (UltraVIEW VoX; PerkinElmer) with a microscope (Eclipse Ti; Nikon) with the Perfect Focus System using an Apochromat 100×, 1.49 NA oil immersion objective (Nikon). Digital images were acquired with an EM charge-coupled device camera (C9100; Hamamatsu Photonics) using Volocity software (PerkinElmer). Z-stacks were collected in steps of 0.1 μm and exposure time of 100 ms. Photobleaching and fluorescence recovery experiments were done as follows: (1) Recorded for 5s at 18.5s fr/s before photobleaching, (2) photobleached for ~4s and (3) recorded recovery of fluorescence for 60 s at a speed of 18.5 fr/s.

### Optical trapping

Single-molecule actomyosin interactions were recorded at room temperature using the three-bead assay geometry in a dual-beam optical-trap system^17^,^18^, with actin adhered to HaloTag-*α*-actinin1*–*coated beads to created bead–actin–bead dumbbells as described^13^. For each dumbbell, the trap stiffness and the system-calibration factor were determined by fitting a Lorentzian function to the power spectrum. The trap stiffness was 0.02–0.03 pN/nm. Dumbbells were pre-tensioned to 1–4 pN and lowered onto the surface of a bead pedestal using a piezoelectric stage controller to scan for actomyosin interactions. Data were filtered at 1 kHz and digitized with a 2-kHz sampling rate. For experiments using the biotinylated Myo1c^3IQ^ construct on nitrocellulose-coated spherical pedestals, single-molecule conditions were verified by diluting the myosin such that approximately one in three pedestals yielded actomyosin interactions.

Spherical SLBs (5 μm dia.) coated with 2% PtdIns(4,5)P_2_, 2% Biotin-PE, 96% DOPC chambers where adhered to planar SLBs of the same lipid composition by flowing the following into a coverslip flow chamber containing the planar SLB: 1 mg/mL streptavidin for 2 min, washed with 100 μL HNa100, 1:10 dilution of lipid coated beads from stock solution in HNa100 for 2 min, washed with KMg25, and followed by the actoMyo1c solution (20–80 pM Actin, 0.2 pM - 360 nM FLMyo1c) in KMg25. Before sealing the chamber with vacuum grease, 3–4 μL of HaloTag *α*-actinin1*–*coated beads were introduced from one side of the chamber. Force relaxation measurements of membrane-bound Myo1c at 1 μM ATP were performed by applying a 125 nm square pulse to both optical traps.

### Laser Trap Data Analysis

Data analysis was performed using in-house software written in LabVIEW. Myosin attachments were detected using the covariance threshold analysis as described^17^,^18^. Traces from each cycle of the square-pulse oscillation experiment were offset so the final relaxation value was zero then normalized to the value of the first point. The distribution of S_5_ (the sum of the five points in the first 2 ms after the force change imposed by the square-wave oscillation (Fig. 2a–e insets)) is variable from dumbbell to dumbbell because of variability in the size of the dumbbell (size of the beads and length of the actin filament) as well as in the stiffness of the trap. In the insets of Fig. 2a–e sample distribution of S_5_ from single datasets are presented while the percentages of the different types of relaxation events in Table 1 were calculated from the whole datasets in each condition. For the analysis of the actoMyo1c-membrane attachment events that relax in a stepwise fashion (Fig. 3) force traces were transformed to displacement traces using Hooke’s law F = *k*_t_·x, where *k*_t_ is the stiffness of the laser trap.

### Coarse-grained model of Myo1c over different substrates

Actin is modeled as a rigid rod constrained to move in one dimension at a fixed height from the surface. The motion of the actin is over-damped as inertial effects are neglected. The actin is embedded in a Langevin solvent^49^, and at each time step, the actin is subject to a random Brownian force due to the thermal motion of the solvent molecules at temperature T, in addition to the forces from the myosin motors and trap. The resulting evolution equation for the position of actin, *x*^*a*^(*t*) is given by


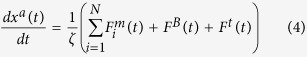


where *ζ* is the longitudinal friction coefficient of the actin dumbbell (Table 2), and is determined experimentally by the product of the relaxation time for an oscillating dumbbell away from any surface (2^nd^ row and 6^th^ column Table 1) and the laser trap stiffness which was 0.02 pN/nm for that particular dataset. In Eq. (4), 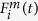 is the force applied by a myosin motor along the actin, 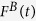 is the random Brownian force, and 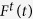 is the force exerted by the optical trap.

Myosin motors are modeled as one-dimensional Hookean springs with stiffness *k*_*m*_ based on optical trap measurements (Table 2), and the force due to the i^th^ motor at a time *t* is given by


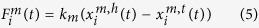


where 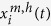 and 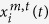 correspond to motor head and tail locations at a given time t, respectively. The trap force is modeled in a similar way, and the net force from the dual trap setup is


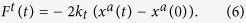


Here, *k*_*t*_ is the compliance of the trap, and *x*^*a*^(0) is the initial location of the left edge of the actin filament (Table 2). The Brownian force is chosen from a Gaussian white noise distribution with zero mean and a variance dictated by the fluctuation-dissipation theorem, i.e.


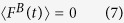



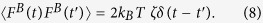


Myosin motor tails are either anchored to the surface (to mimic nitrocellulose surface) or allowed to diffuse (to mimic the lipid membrane). The motors are randomly distributed over the surface, and if the tail is anchored, the tail positions are given by *x*^*m,t*^(*t*) = *x*^*m,t*^(0). If the tail is diffusive, the equation of motion is given by


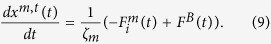


Here *ζ*_*m*_ is the friction coefficient of the myosin tail and *F*^*B*^(*t*) once again denotes the Brownian force. Both actin and myosin equations of motion are integrated using an Euler scheme with a time step of 0.5 μs. The friction coefficients are calculated using Einstein relation from the diffusion coefficient.

Myosin motors detach from the actin in a force dependent manner, based on the off rate, *k*_off_(*F*), given by


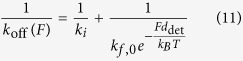


where *k*_*i*_ is the force-independent rate, *k*_f,0_ is the unloaded force-dependent rate, *d*_det_ is the detachment distance parameter^13^ (Table 2). Myosins can rebind to actin based on their on-rate^17^, *k*_on_, determined using the force dependent off rate and the duty ratio^13^ given by


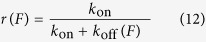


During a simulation time step, motors attach or detach stochastically based on these rates. Myosin tails also detach from the surface, in the presence of the lipid membrane coated pedestal. The detachment rate is given by 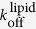, measured for a single myosin motor in the absence of actin (Table 2). The unbound tails are attached to the surface immediately at zero force, i.e. the tail and head positions of the corresponding i^th^ motor are the same 
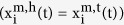
. Although the membrane-detachment rate is likely to be force-sensitive^16^, the dependence of this rate on the direction of the applied force is not known and an analytical expression that describes the force sensitivity is not available.

## Additional Information

**How to cite this article**: Pyrpassopoulos, S. *et al*. Force Generation by Membrane-Associated Myosin-I. *Sci. Rep*. **6**, 25524; doi: 10.1038/srep25524 (2016).

## Supplementary Material

Supplementary Information

Supplementary Movie S1

Supplementary Movie S2

Supplementary Movie S3

Supplementary Movie S4

Supplementary Movie S5

Supplementary Movie S6

## Figures and Tables

**Table 1:** Numbers and percentages of the different types of relaxation events during oscillation experiments of an actin dumbbell and the calculated values of *τ* (ms), *τ*_*Myo1c*_ (ms), *γ*_*Myo1c*_ (pN·s/nm), *D*_*Myo1c*_ (μm^2^/s) (Figs 1, 2, 3, see also main text for details).

			#events	%	*τ*^1^ (ms)	*τ*_*Myo1c*_^2^ (ms)	*γ*_*Myo1c*_^3^ (pN·s/nm)	*D*_*Myo1c*_^4^ (μm^2^/s)
**Dumbbell oscillation away from pedestal**	S_5_ ≤ μ + 2.5σ	Exponential relaxations	2393	99.6	0.79			
	S_5_ >μ + 2.5σ	Exponential relaxations	9	0.4	1.2			
		Stepwise relaxations	0	0				
**Dumbbell oscillation in contact with lipid-coated pedestal**	S_5_ ≤ μ + 2.5σ	Exponential relaxations	3927	99.5	1.0			
	S_5_ >μ + 2.5σ	Exponential relaxations	21	0.5	1.7			
		Stepwise relaxations	0	0				
**Dumbbell oscillation in contact with lipid-coated pedestal and 0.2 pM Myo1c**	S_5_ ≤ μ + 2.5σ	Exponential relaxations	2313	93.3	0.82	0.78	0.023·10^−3^	0.18
	S_5_ >μ + 2.5σ	Exponential relaxations	21	0.9	1.6			
		Stepwise relaxations	144	5.8				
**Dumbbell oscillation in contact with lipid-coated pedestal and 0.6 pM Myo1c**	S_5_ ≤ μ + 2.5σ	Exponential relaxations	3654	90.4	0.68	0.92	0.028·10^−3^	0.15
	S_5_ >μ + 2.5σ	Exponential relaxations	66	1.6	1.6			
		Stepwise relaxations	322	8				
**Dumbbell oscillation in contact with lipid-coated pedestal and 1.9 pM Myo1c**	S_5_ ≤ μ + 2.5σ	Exponential relaxations	2986	63.5	0.84	0.86	0.024·10^−3^	0.17
	S_5_ >μ + 2.5σ	Exponential relaxations	123	2.6	1.7			
		Stepwise relaxations	1593	33.9				

^1^*τ* was calculated by fitting the ensemble average of the corresponding traces to a single exponential decay function.

^2^*τ*_*Myo1c*_ was calculated as the difference between the two values of the previous column for each condition (see equation 2 in main text).

^3^*γ*_*Myo1c*_ was calculated using the values of *τ*_*Myo1c*_ from previous column and equation 2.

^4^*D*_*Myo1c*_ was calculated using the values of *γ*_*Myo1c*_ from previous column and Einstein’s equation (equation 3 of main text).

**Table 2:** Parameters used in the simulations.

Parameter	Value
Actin length, *L*_*actin*_	10 μm
Actin initial position, x_0_	0 μm
Actin dumbbell friction coefficient, 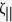	1.58 × 10^−5^pN s/nm
Pedestal length, L_pedestal_	705 nm
Trap compliance, *k*_t_	0.02pN/nm
Detachment rate from lipid, 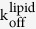	2s^−1^
Attachment rate to actin, k_on_ (ref. 48)	0.7 s^−1^
Motor compliance, *k*_m_ (ref. 14)	0.2pN/nm
Time step, δ	5 × 10^−7^ s
Temperature, T	293 K
Force independent detachment rate from actin, k_i_ (ref. 13)	5.6 s^−1^
Distance parameter, d_det_ (ref. 13)	5.2 nm
Force dependent rate in the absence of load, k_f0_ (ref. 13)	29 s^−1^
Force dependent detachment rate from actin, 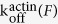	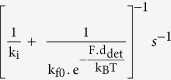

**Table 3:** Average force generated in three different regions on and the entire length of the pedestal by myosin motors for different substrates at different motor densities.

	Region	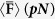	<n>	<F_tot_>(*pN*)
Lipid (N = 69.04)	*Left*	0.246	3.89	0.957
	*Middle*	0.0076	62.9	0.478
	*Right*	−0.0340	2.26	−0.0768
	*Total*	0.0197	69.0	1.36
Lipid (N = 123.61)	*Left*	0.287	6.61	1.90
	*Middle*	0.0074	113	0.838
	*Right*	−0.0239	3.76	−0.0899
	*Total*	0.0214	124	2.65
NC(N = 2.14)	*Left*	0.752	0.160	0.120
	*Middle*	0.713	1.81	1.29
	*Right*	0.732	0.170	0.125
	*Total*	0.718	2.14	1.54
NC(N = 3.56)	*Left*	1.04	0.180	0.187
	*Middle*	1.04	3.19	3.33
	*Right*	1.05	0.190	0.200
	*Total*	1.04	3.56	3.71

These regions are the *left* and *right* boundary regions, corresponding to the first 8 nm from the outermost points of the pedestal, and the *middle* region in between. *Total* region is the entire pedestal length. Here N is the average number of motors bound to actin, <n> is the average number of bound motors to actin in a given region, 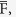
denotes the average force of a single myosin during *τ*_on_, the time average of the term 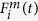 in Eq. 4 (see Methods section) during actin attachment, 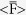 is this mean force averaged over all the myosins in this region. After these averages are obtained from each simulation, the results are also averaged over an ensemble of 50 runs. The total average force, <F_tot_>, is calculated from the product of <n> and 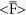. We want to mention that the calculation method of these average values is different from the calculation method in Fig. 6.
