# Supplementary material for: Force Generation by Membrane-Associated Myosin-I
Source: Sci Rep. 2016 May 9;6:25524. doi: 10.1038/srep25524 (PMC4860596; doi:10.1038/srep25524)
Supplement: Supplementary Information [file srep25524-s1.pdf]

Supplementary Information

Force Generation by Membrane-Associated Myosin-I

Serapion Pyrpassopoulos[1], Göker Arpağ[2], Elizabeth A. Feeser[1], Henry Shuman[1], Erkan Tüzel[2*], and E. Michael Ostap[1*]

[1]The Pennsylvania Muscle Institute and Department of Physiology
Perelman School of Medicine at the University of Pennsylvania, Philadelphia PA 19104-6085

[2]Department of Physics
Worcester Polytechnic Institute, Worcester, Massachusetts.

Corresponding Authors:

[*]Erkan Tüzel
Department of Physics
Worcester Polytechnic Institute
100 Institute Road, Olin Hall
Worcester, MA, 01609
Email: tuzel@mailaps.org

[*]E. Michael Ostap
Department of Physiology
Perelman School of Medicine at the University of Pennsylvania
700A Clinical Research Building
415 Curie Blvd.
Philadelphia, PA 19104-6085
Phone: 215-573-9758
Email: ostap@mail.med.upenn.edu

Supplementary Methods

Stability of attachment of lipid coated spherical pedestals on planar SLBs.

We checked the attachment stability of lipid coated spherical pedestals (dia. = 5 μm) on planar supported lipid bilayers via streptavidin (see Materials and Methods) by ensuring the pedestal did not move under loads encountered during an experiment. We centered the laser trap on the spherical pedestal and applied a square wave pulse that produced pulling forces on the spherical pedestal similar to the forces developed during the actin dumbbell oscillation experiments (≤ 5 pN). The average force traces over multiple cycles when (a) a lipid coated pedestal was attached on a planar lipid bilayers (black trace) (b) a spherical pedestal was immobilized on glass coverslip under a nitrocellulose film (red trace) and (c) a spherical pedestal was free in solution (blue trace) are shown in Supplementary Fig. 1. As can be seen our immobilization strategy of lipid coated spherical pedestals is indistinguishable from the standard immobilizing strategy under a thin nitrocellulose film.

Parsing stepwise from the non-stepwise relaxation events during an actoMyo1c attachment of the dumbbell with the lipid membrane.

After the initial S_5-based identification of actoMyo1c interactions, we further processed the data to separate stepwise form non-stepwise relaxations. The stepwise traces take more time to relax to the baseline force than exponentially decreasing interactions, so additional summing thresholds were used to further separate the data. Supplementary Fig. 2 illustrates the use of the sum of the first 12 points (S_{12}) to identify stepwise

interactions. The efficient separation was verified by graphical displays for each data set (Supplementary Fig. 2).

Diffusion of SLB-Bound Myo1c

Actin (2 μM) was polymerized for 30 min in KMg25 at room temperature in the presence of 2 μM Alexa Fluor 488 phalloidin (Life Technologies) and 400 nM gelsolin. The proteins were dialyzed overnight against KMg25 to remove free ATP and then sonicated for 5 min to shear filaments. Observation chambers were made from glass coverlips with planar SLBs consisting of (a) 2% PtdIns(4,5)P_2 and 98% DOPC, (b) 0.2% PtdIns(4,5)P_2 and 99.8% DOPC, or (c) 100 % DOPC [1]. Actin (1 – 0.1 nM) and Myo1c (0.1 - 0.01 nM) in KMg25, supplemented with apyrase VII, 10 μM calmodulin, 1 mg/ml glucose, 192 U/ml glucose oxidase [Sigma] and 48 mg/ml catalase [Roche] was introduced in the Chamber. Sequential images were recorded using TIRF microscopy (560 nm laser) on an inverted Leica microscope at 20-100 frames/s. The density of diffusing filaments per field of view was low (~ 10 filaments per field of view 77 x 99 μm). Filaments were small enough to be diffraction limited. Filaments exhibit a transient behavior diffusing for a period of time and then dissociating, while others could be seen coming in and out of the TIRF plane within 1 frame, supporting the sparse decoration of actin with Myo1c. Two-dimensional diffusion trajectories of fluorescent actin filaments were measured using Image J tracking plugin written by Yale E. Goldman[2] (Supplementary Fig. 4). For each track we calculated the displacements Δr for successive frames (Δt = 1 frame), the mean square displacement (MSD) $\langle \Delta r^2 \rangle$ and then the diffusion coefficient D from the equation [3],

$$D = \frac{\langle \Delta r^2 \rangle}{4 \cdot \Delta t}$$

The distribution of the calculated values for *D* is plotted in Supplementary Fig. 4 and the average value is $\langle D \rangle$ = 0.33 ± 0.37 μm^2/s. $\langle \Delta r^2 \rangle$ values were also calculated for different time steps Δt and the corresponding plots are linear within error as expected for diffusive behavior (Supplementary Fig. 4). Based on this average value $\langle D \rangle$ = 0.33 ± 0.37 μm^2/s and the Maxwell model (see Maxwell model for the relaxation of a dumbbell), the expected lifetime for relaxation of load applied to membrane bound Myo1c is in the range 0.41 – 0.62 ms for laser trap stiffness *k* in the range 0.03 – 0.02 pN/nm, respectively.

Maxwell model for the relaxation of a dumbbell.

We used the Maxwell Model to describe the relaxation of the force that a pre-tensioned actin dumbbell experiences upon an abrupt change in the positions of the two laser traps, i.e. a Hookean spring with elasticity, *k*, connected in series with a dashpot of damping coefficient, γ [4]. The spring element corresponds to the stiffness of the laser trap (*k* = 0.02 - 0.03 pN/nm), and the dashpot element corresponds to the viscous force experienced by the dumbbell due to the aqueous environment. The relaxation of the force is given by the equation

$$F(t) = F(0) \cdot e^{-t/\tau}$$

where $\tau = \gamma/k$ is the relaxation lifetime, *k* is the stiffness of the laser trap and γ the drag coefficient of the actin dumbbell [4]. To estimate the relaxation lifetime we therefore need to calculate the Stokes' drag coefficient for the actin dumbbell. We assumed that the drag coefficients of the components of the actin dumbbell are additive:

$$\gamma_{dbblll} \cong 2\cdot\gamma_{bead} + \gamma_{actin} = 2\cdot 6\pi\eta r + 2\pi\eta L/[\ln(L/2R) - 0.2] = 2.7\cdot 10^{-5} \text{ pN}\cdot\text{s/nm}$$

where η = 0.001 N·s·m^{-2} is the viscosity of water, r = 0.5 μm is the radius of the bead and the actin filament is approximated as a cylinder of diameter $2\cdot R$ = 10 nm and length L = 10 μm. The relaxation lifetime of the free dumbbell (in the absence of membrane attachment) is expected to be

$$\tau_{dbbll} \cong 1.4 - 0.91 \text{ ms for } k = 0.02 – 0.03 \text{ pN/nm}.$$

Due to hydrodynamic screening [5], the above coefficient is expected to be an overestimation of the true value.

Supplementary Figures

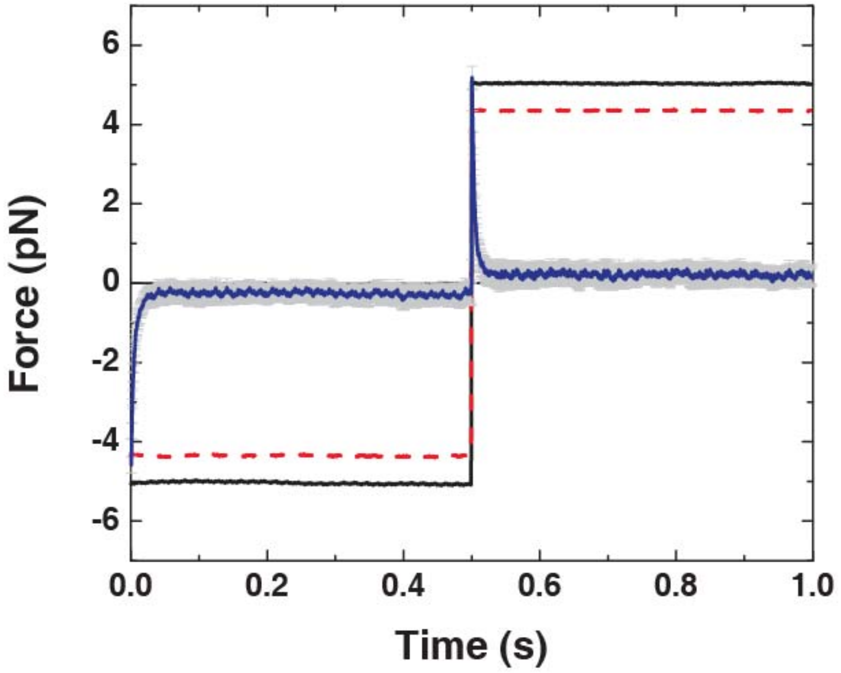

Supplementary Figure 1. Stability of lipid coated pedestals.

Plot of the average force traces on spherical pedestals (5 μm in diameter) over multiple square pulse oscillation cycles (T_{osc} = 1s) when the laser trap is centered at the spherical pedestal for three different cases: 1) pedestal is lipid coated and attached via streptavidin to a planar lipid bilayer (black color) 2) pedestal is immobilized under a nitrocellulose film on a glass coverslip (red color) and (3) pedestal is free in solution (blue color; error bars (standard deviation) are shown with gray color).

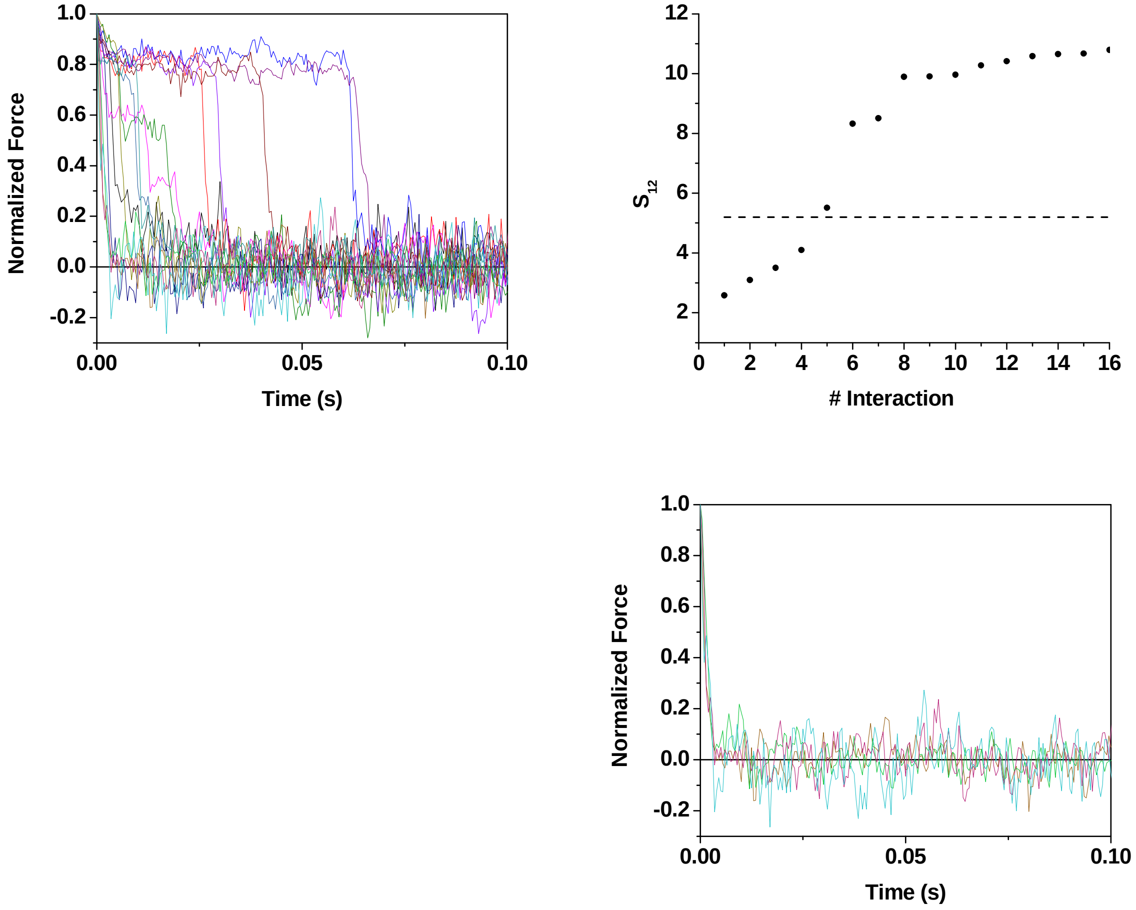

Supplementary Figure 2. Separating stepwise from exponentially relaxing events.

(a) Sample traces of 16 events identified as attachment events during actoMyo1c attachment to membrane-supported pedestal at 1μM ATP. The traces were acquired in the presence of 0.2 pM Myo1c and selected based on the criterion $S_5 > \mu+2.5\cdot\sigma$ (see Main Text).

(b) Values for the sum S_{12} of the first 12 points, sorted in ascending order, for the 16 traces shown in panel (a). The dashed line indicates a threshold value (5.2) for S_{12} that separates the stepwise form the non-stepwise relaxation events. (c) The 4 traces out of the 16, for which $S_{12} < 5.2$.

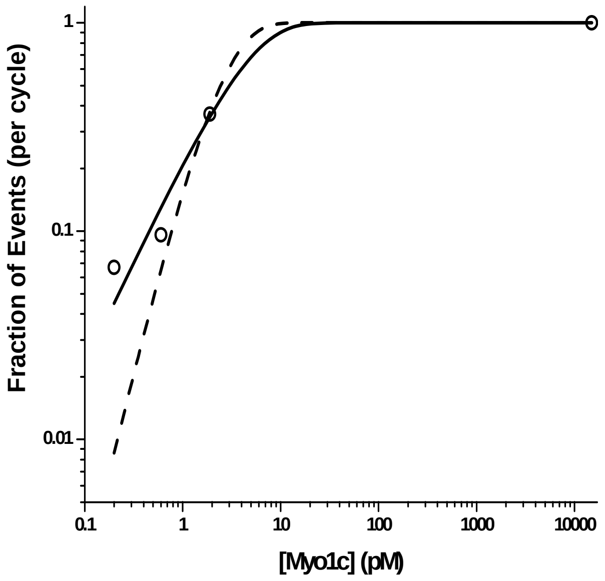

Supplementary Figure 3. Probability of actoMyo1c-membrane attachments on lipid coated pedestals as a function of Myo1c concentration.

The fraction of actoMyo1c membrane attachments over the total number of oscillation cycles as a function of [Myo1c] at 1 μM ATP. We adopted the approach from Mallik et al. [6]. The first three points are calculated based on Table 1 and the last point for [Myo1c] = 15 nM represents the saturated state. The solid curve is a fit to single-molecule Poisson distribution $P(1;\mu) = 1- P(0;\mu) = 1-\exp(-\mu) = 1-\exp(-[Myo1c]/b)$ with b = 4.3 pM and reduced $\chi^2 = 0.56 \cdot 10^{-3}$. The dashed curve is a fit to a two molecule Poisson distribution $P(2;\mu) = 1- P(0;\mu) – P(1;\mu) = 1- \exp(-\mu)-\mu \cdot \exp(-\mu)$ with b = 1.5 pM and reduced $\chi^2 = 1.5 \cdot 10^{-3}$. For the data at 0.2 and 0.6 pM Myo1c the fraction of interaction is less than 0.1 and based on the single-molecule fitting the probability of an interaction to be due to two-molecules is < 0.5%.

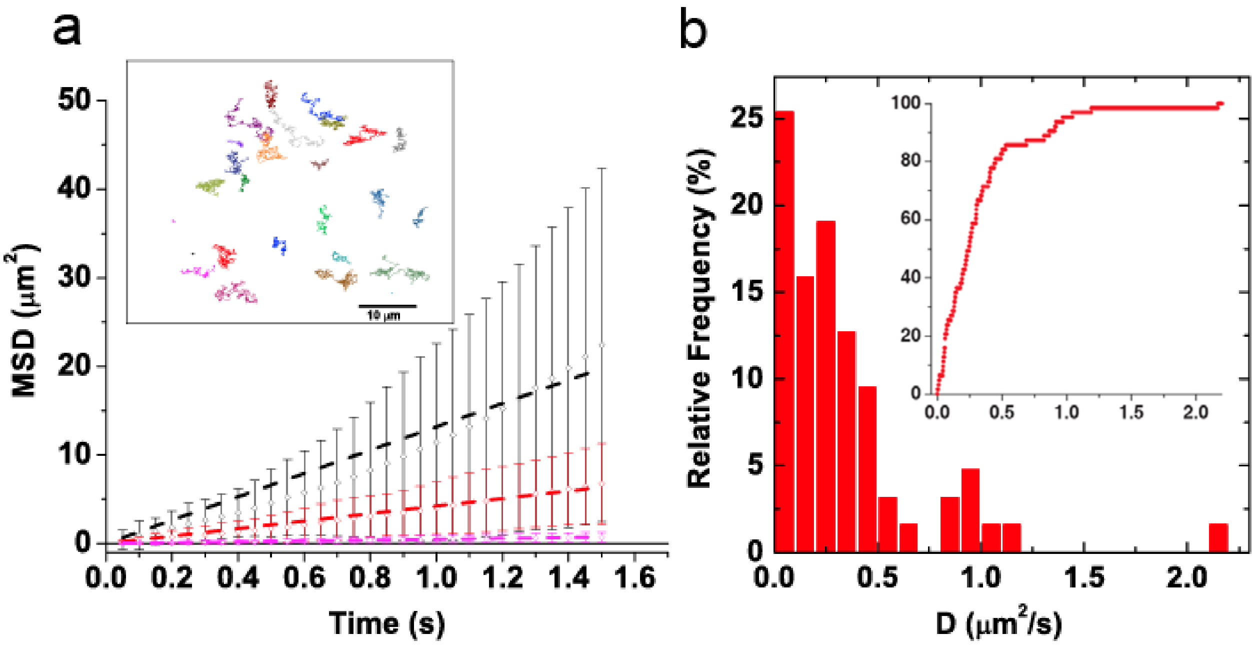

Supplementary Figure 4. Diffusion of actoMyo1c on supported lipid bilayers.

(a, inset) Tracks of diffraction-limited fluorescent actin filaments bound to 2% PtdIns(4,5)P_2 planar-supported-lipid-bilayers via Myo1c acquired using TIRF microscopy. (a, main plot) Representative plots of the mean square displacement $<\Delta r^2>$ for three different tracks as a function of the time step window Δt (0.05 s – 1.5 s). Dashed lines are error-weighted fits to a linear function y = A·x. (b) Frequency distribution of the individual diffusion coefficient values D_{Myo1c} for membrane-bound Myo1c as obtained from 63 different tracks of fluorescent actin filament observed using TIRF microscopy. (Inset) The same data plotted as a cumulative distribution.

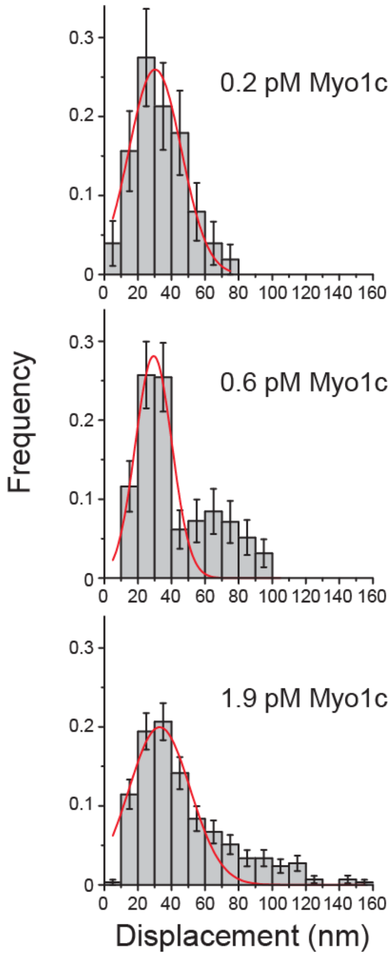

Supplementary Figure 5. Step-size distribution of stepwise interactions.

Frequency distributions for the size of stepwise interactions (see Fig. 3 of main text) at 1 μM ATP and 0.2, 0.6 and 1.9 pM of Myo1c. Error bars were estimated from 1000 bootstrap cycles and the red trace is an error-weighted fit of each distribution to a Gaussian function.

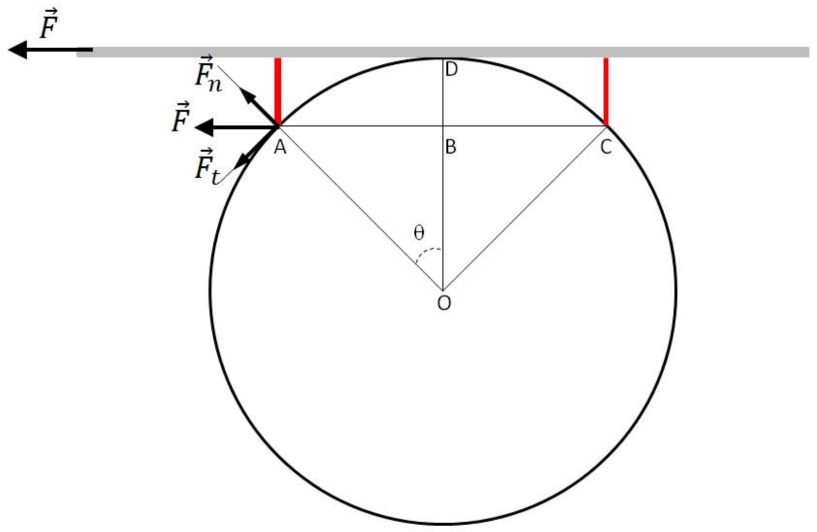

Supplementary Figure 6. Cartoon outline (not drawn to scale) of actin-filament pedestal geometry. The actin filament is indicated by the gray bar, Myo1c by the red bar and the circle represents the spherical immobilized pedestal. The length of the actin filament that can interact with the spherical surface via myosin1c is

$$|AC| = |BD| \sqrt{\left(2\frac{|OA|}{|BD|} - 1\right)},$$

where BD = 25 nm is the length of Myo1c [7,8] and OA = 2500 nm is the radius of the spherical pedestal. For the above numerical values, the equation gives AC = 705 nm, which is the length of the pedestal in our one dimensional computer simulations (Supplementary Fig 7).

The angle θ then is found equal to 8.11° or 0.1415 rad and the length of the arc ADC is 708 nm. For this value of θ, the force component F_n will be negligible since $\sin\theta = 0.14$ and $F_n = 0.14 \cdot F$.

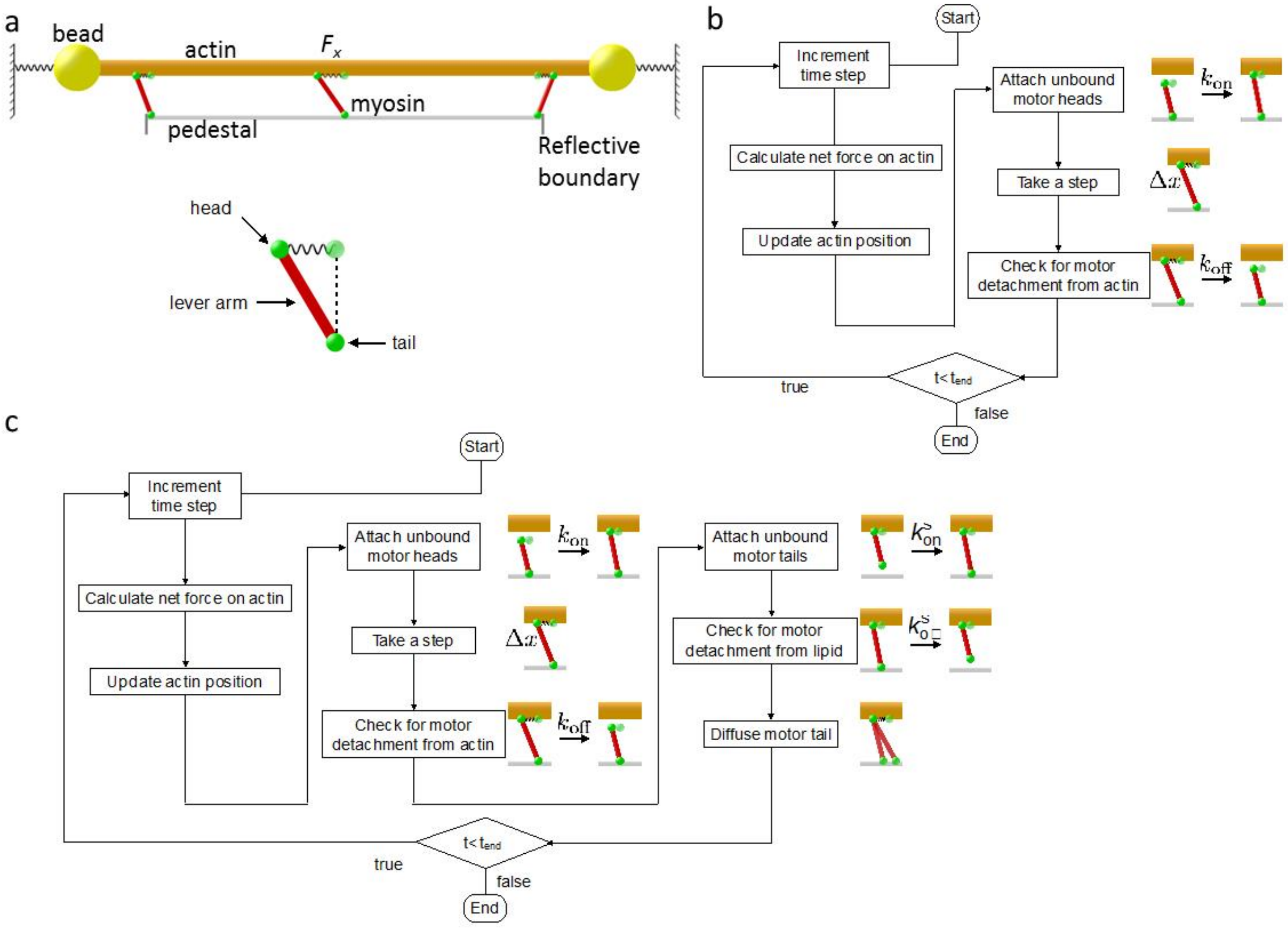

Supplementary Figure 7. A sketch of the model geometry and flowchart of the simulations.

Schematic of the coarse-grained model is shown in panel (a). Actin is modeled as a rigid rod with two beads at the ends where the trap force is applied. Myosin motors are modeled as one-dimensional springs where the only force, F_x, is along the axis of the actin. The pedestal is modeled as a finite length one-dimensional segment, with motor tails either fixed (representing nitrocellulose coated surface) or allowed to diffuse (representing lipid coated surface) along the axis of the pedestal. Reflective boundary conditions are applied at the ends of the pedestal, i.e. when a motor tail tries to move out of the pedestal, it will remain at the same position. In (b) and (c) flow charts of the simulations are shown for the nitrocellulose and lipid coated pedestal versions of the model, respectively.

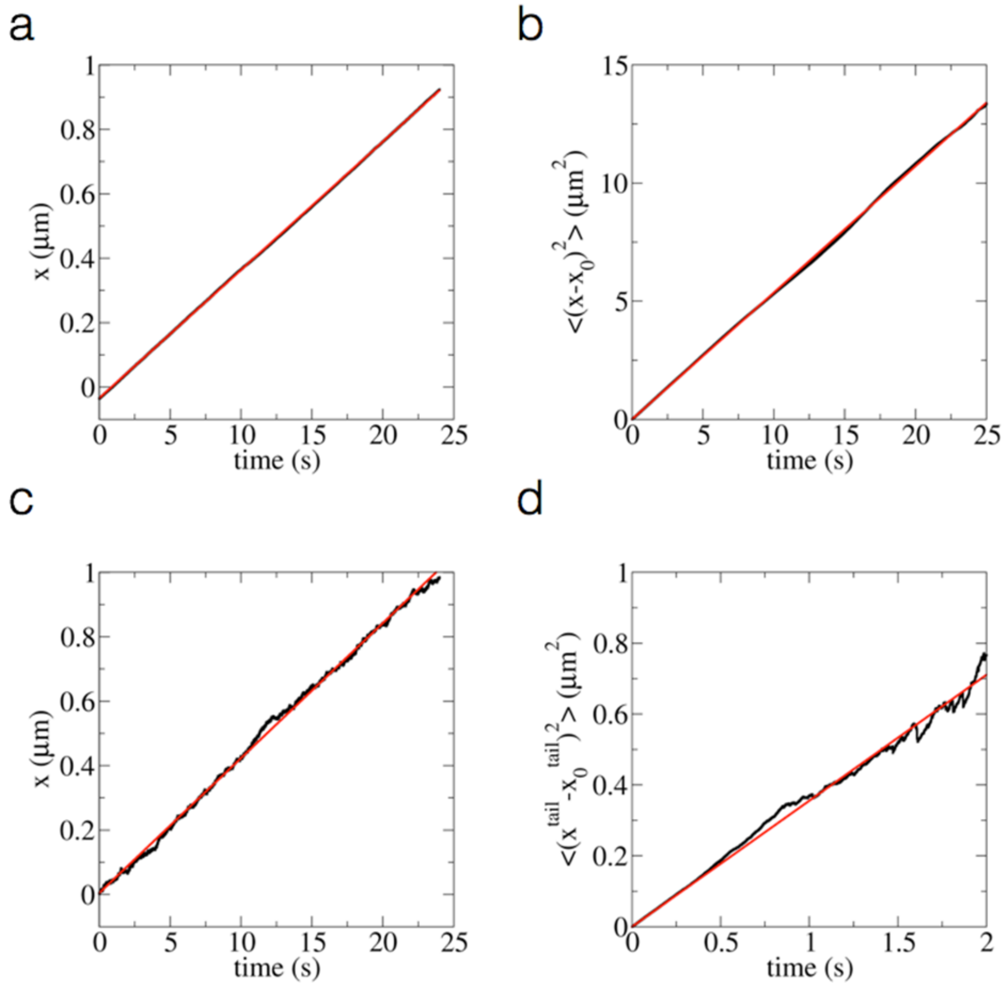

Supplementary Figure 8. Position and MSD plots from the simulations.

In panels (a) and (c), the position of actin as a function of time is shown in the absence of the optical trap for the motor density ρ = 100 motors/μm of pedestal, for nitrocellulose- and lipid-coated pedestal surfaces, respectively. Data are ensemble averaged over 50 realizations, and the fits (red lines) give actin gliding velocities of v = 39.8 and 41.9 nm/s, for (a) and (c), respectively. In (b), the mean squared displacement (MSD) of actin is shown as a function of time in the absence of motors and the optical trap, averaged over 500 realizations. The fit (red line) gives a diffusion coefficient of D = 0.268 μm^2/s in excellent agreement with the experimentally measured D = 0.260 μm^2/s ($D = k_B T/\zeta$, where $\zeta = 1.58 \cdot 10^{-5}$ pN·s/nm is the longitudinal friction coefficient of the actin dumbbell (see Methods and Table2) and $k_B T$ = 4.05 pN·nm). In (d), the MSD of freely diffusing motors on the lipid-coated surface is shown, averaged over 500 motors. The fit (red line) gives a diffusion coefficient of D = 0.178 μm^2/s in excellent agreement with the experimentally obtained average diffusion coefficient ($\langle D \rangle$ = 0.170 μm^2/s).

Supplementary Movies

Supplementary Movie 1. Confocal imaging of spherical pedestals (dia. = 5 μm) coated with lipid bilayer (0.1% LRPE-2% PtdIns(4,5)P_2 – 2% BiotinPE – 95.9% DOPC) and anchored via streptavidin on a planar lipid bilayer of similar composition. Sequential frames were taken every 0.1 μm along the Z-axis from top to bottom.

Supplementary Movie 2. Confocal imaging of fluorescence recovery after photobleaching a small area on the top of spherical (dia. = 5 μm) supported lipid bilayers containing 0.1% LRPE.

Supplementary Movie 3. A sample movie from the simulations showing the actin dumbbell over a nitrocellulose substrate at a high density of myo1c motors (average number of actin-bound motors, N = 3.56, as shown in Table 3). The trap force is shown via springs connected to the beads (yellow). Vertical (dark gray) bars illustrate the reflective boundary conditions of the pedestal (shown in gray). Motors are color-coded to show the force exerted at a given time. Motors with forces larger than 2 pN are shown in green. Plots below show the number of actin-bound motors (N) and the trap force (F) with the probability histogram as a function of time, respectively.

Supplementary Movie 4. A sample movie from the simulations showing the actin dumbbell over a lipid coated substrate at a high density of myo1c motors (average number of actin-bound motors, N = 123.61, as shown in Table 3). The trap force is

shown via springs connected to the fluorescent beads (yellow). Vertical (dark gray) bars illustrate the reflective boundary conditions of the pedestal (shown in gray). Motors are color-coded to show the force exerted at a given time. Motors with forces larger than 2 pN are shown in green. Plots below show the number of actin-bound motors (N) and the trap force (F) with the probability histogram as a function of time, respectively. For brevity, only 25% of the motors are shown.

Supplementary Movie 5. A sample movie from the simulations showing the actin dumbbell over a nitrocellulose substrate at a low density of myo1c motors (average number of actin-bound motors, N = 2.14, as shown in Table 3). The trap force is shown via springs connected to the fluorescent beads (yellow). Vertical (dark gray) bars illustrate the reflective boundary conditions of the pedestal (shown in gray). Motors are color-coded to show the force exerted at a given time. Motors with forces larger than 2 pN are shown in green. Plots below show the number of actin-bound motors (N) and the trap force (F) with the probability histogram as a function of time, respectively.

Supplementary Movie 6. A sample movie from the simulations showing the actin dumbbell over a lipid coated substrate at a low density of myo1c motors (average number of actin-bound motors, N = 69.04, as shown in Table 3). The trap force is shown via springs connected to the fluorescent beads (yellow). Vertical (dark gray) bars illustrate the reflective boundary conditions of the pedestal (shown in gray). Motors are color-coded to show the force exerted at a given time. Motors with forces larger than 2 pN are shown in green. Plots below show the number of actin-bound motors (N) and the

trap force (F) with the probability histogram as a function of time, respectively. For brevity, only 25% of the motors are shown.

1. Pyrpassopoulos, S., Feeser, E.A., Mazerik, J.N., Tyska, M.J. & Ostap, E.M. Membrane-bound myo1c powers asymmetric motility of actin filaments. *Curr Biol* **22**, 1688-92 (2012).
2. Ross, J.L., Wallace, K., Shuman, H., Goldman, Y.E. & Holzbaur, E.L. Processive bidirectional motion of dynein-dynactin complexes in vitro. *Nat Cell Biol* **8**, 562-70 (2006).
3. Saxton, M.J. Single-particle tracking: the distribution of diffusion coefficients. *Biophys J* **72**, 1744-53 (1997).
4. Howard, J. *Mechanics of Motor Proteins and the Cytoskeleton*, 367 (Sinauer Associates, Inc., Sunderland, 2001).
5. Phillies, D.J. Translational drag coefficients of assemblies of spheres with higher-order hydrodynamic interactions. *J Chem Phys* **81**, 4046-4052 (1984).
6. Mallik, R., Petrov, D., Lex, S.A., King, S.J. & Gross, S.P. Building complexity: an in vitro study of cytoplasmic dynein with in vivo implications. *Curr Biol* **15**, 2075-85 (2005).
7. Munnich, S., Taft, M.H. & Manstein, D.J. Crystal structure of human myosin 1c--the motor in GLUT4 exocytosis: implications for Ca2+ regulation and 14-3-3 binding. *J Mol Biol* **426**, 2070-81 (2014).
8. Lu, Q., Li, J., Ye, F. & Zhang, M. Structure of myosin-1c tail bound to calmodulin provides insights into calcium-mediated conformational coupling. *Nat Struct Mol Biol* **22**, 81-8 (2015).
